# Rheumatoid arthritis associated interstitial lung disease: diagnostic accuracy of lung ultrasound compared to chest high resolution computed tomography

**DOI:** 10.3389/fmed.2026.1774934

**Published:** 2026-02-20

**Authors:** Rim Dhahri, Islem Mejri, Ichrak Ouslati, Soumaya Boussaid, Lobna Ben Ammar, Hiba Ben Ayed, Tasnim Znegui, Refka Jebri, Chirine Moussa, Zied Moatamri, Khalil Amri, Moncef Aloui, Imen Gharsallah

**Affiliations:** 1 Department of Rheumatology, Military Hospital of Instruction, Tunis, Tunisia; 2 Faculty of Medicine of Tunis, University of Tunis el Manar, Tunis, Tunisia; 3 Department of Pneumology, Military Hospital of Instruction, Tunis, Tunisia; 4 Department of Rheumatology, Rabta Hospital, Tunis, Tunisia; 5 Department of Orthopedic Surgery, Military Hospital of Instruction, Tunis, Tunisia

**Keywords:** high resolution computed tomography, interstitial lung disease, lung disease, lung ultrasound, rheumatoid arthritis

## Abstract

**Introduction:**

Interstitial lung disease (ILD) affects up to 60% of patients with rheumatoid arthritis (RA). Chest high-resolution computed tomography (HRCT) remains the gold standard for diagnosis; however, it involves radiation exposure. Lung ultrasound (LUS) is a safe and accessible tool, but it has not yet been validated for ILD diagnosis in RA.

**Methods:**

We conducted a prospective, single-center, cross-sectional study including patients followed for RA. Exclusion criteria were pregnancy, history of heart failure, acute cardiac decompensation, respiratory symptoms within the last 3 months, and pneumonia within the past month. All included patients underwent both LUS and chest HRCT. The diagnostic performance of LUS was assessed using HRCT as the reference standard.

**Results:**

A total of 73 patients were included (18 men and 55 women), with a mean age of 55 ± 12 years. The mean Disease Activity Score (DAS28-ESR) was 3.47 ± 1.40. Chest HRCT identified ILD in 28.8% of patients. According to the semi-quantitative ultrasound score, interstitial involvement was detected in 21.9% of patients. The sensitivity of LUS was 59.1%, and the specificity was 94.1%. Receiver operating characteristic (ROC) curve analysis showed an area under the curve (AUC) of 0.813. The optimal cutoff was 5 B-lines, yielding a sensitivity of 63.6% and a specificity of 94.1%.

**Discussion:**

LUS demonstrated good diagnostic performance for ILD detection in RA patients, with high specificity. These findings suggest that LUS may represent a useful screening tool to identify patients requiring further evaluation with HRCT.

## Introduction

Ultrasound represents a cost-effective, radiation-free, and available diagnostic tool. It has been applied in the detection of extra-articular manifestations in chronic inflammatory rheumatic diseases. Notably, diaphragmatic ultrasonography has shown particular utility in assessing respiratory involvement in patients with spondylarthritis (SpA) (1), Speckle-tracking echocardiography (STE) enables early cardiovascular risk assessment through the detection of myocardial impairment in patients with rheumatoid arthritis (RA) (2). Emerging studies are evaluating its role in the diagnosis of interstitial lung disease (ILD) in RA. ILD affects up to 60% of RA patients, with severe complications occurring in approximately 10% of cases (3). Thus, early diagnosis is essential, especially with the emergence of antifibrotic agents that have improved prognosis (4). However early diagnosis remains challenging, as clinical signs may be absent in patients with ILD (5), Standard radiography is an accessible but irradiating examination with low sensitivity (6).

Pulmonary function testing (PFT) is an essential tool for assessing the progression of ILD and guiding subsequent therapeutic decisions, but it has low sensitivity for detecting early, mild to moderate interstitial involvement (7, 8).

Chest high resolution computed tomography (HRCT) remains the gold standard for diagnosis (9), however, it poses challenges due to its high radiation exposure and elevated cost.

Despite its safety, lung ultrasound (LUS) is not yet validated for the diagnosis of ILD in RA, and currently, no consensus ultrasound protocol exists. Our study aims to assess the performance of LUS in diagnosing ILD in RA.

## Methods

We conducted a prospective, single-center cross-sectional study. Eligible patients were consenting adults (age >18 years), diagnosed with RA according to the ACR-EULAR criteria (10). Pregnant women, patients with a history of heart failure, acute cardiac decompensation, recent respiratory symptoms within the last 3 months, or a recent pneumonia episode within the past month were excluded.

### Study design

Eligible patients were consecutively recruited on the day of their appointment at the Rheumatology Department of the Principal Military Teaching Hospital of Tunis. A rheumatologist collected RA-related clinical data, evaluated the presence or absence of dyspnea, and assessed exercise-induced desaturation during the 6-min walk test (6MWT) desaturation was defined as a ≥ 4% drop in baseline oxygen saturation. Spirometry was requested if no test had been performed within the previous 3 months; the median time to spirometry was 10 days (IQR: 5–20 days), ranging from 1 to 60 days. A chest HRCT was ordered for patients whose previous HRCT was older than 1 year. Each patient was then referred to the Pulmonology Department to undergo LUS on the same day, performed by a pulmonologist with 8 years of experience. The median interval between the chest HRCT and LUS was 30 days (IQR: 15–100), with a minimum interval of 3 days and a maximum of 300 days.

### LUS

LUS was performed using the EDAN ACCLARIX AX3 device. A low-frequency convex probe (2.5–5 MHz) was used with the frequency set at 5 MHz.

**Figure 1:**
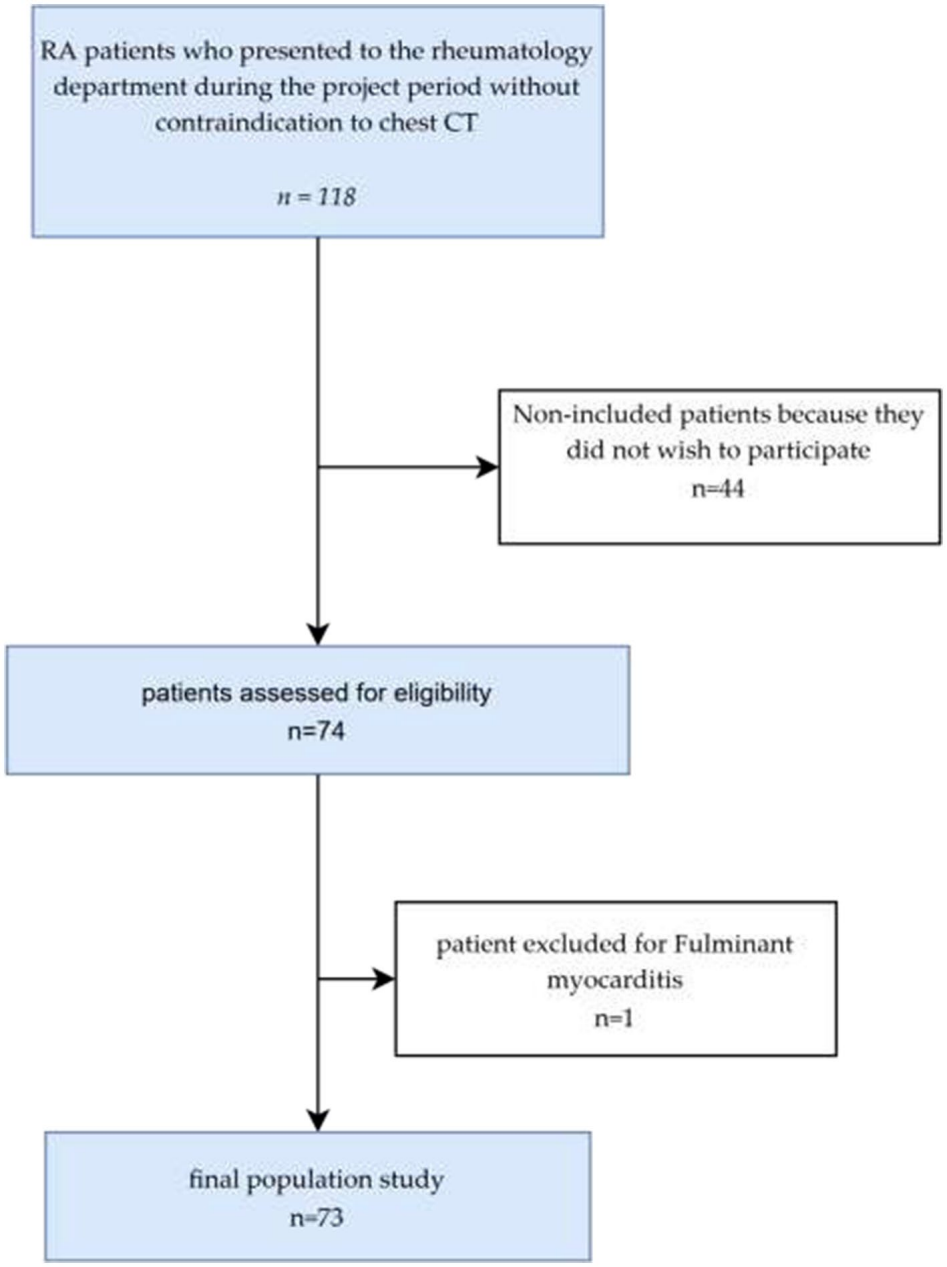
ROC curve assessing the diagnostic performance of LUS for the detection of ILD.

The 12-zone scanning protocol was applied for the examination (11). The thorax was systematically divided, on both the right and left sides, into three regions: anterior, lateral, and posterior. Each region was then subdivided into upper and lower segments, resulting in a total of 12 zones, numbered from T1 to T12.

The 12-zone protocol was chosen as it ensures a balanced assessment of anterior, lateral, and posterior thoracic areas on both sides (12), these zones have been validated as predictive of changes related to interstitial involvement (8). Moreover, the operator who performed the LUS was experienced with this protocol.

Ultrasound signs suggestive of interstitial involvement were assessed, including B-lines and pleural irregularities.

B-lines are vertical, hyperechoic reverberation artifacts that arise from the pleural line and extend to the bottom of the screen without fading (12).

The presence of three or more B-lines per scan defines a B-pattern and suggests interstitial involvement (13).

We used a semi-quantitative scoring system to classify ultrasound-detected interstitial involvement (11). Based on the total number of B-lines, we applied the following semi-quantitative classification: score 0 (≤5) indicated no interstitial involvement, score 1 (6–15) mild involvement, score 2 (16–30) moderate involvement, and score 3 (≥30) severe involvement. Pleural line irregularities defined as a discontinuity of the bright horizontal pleural line were also assessed, as they are suggestive of subpleural fibrosis (12).

### Chest HRCT

Chest HRCT was performed without contrast injection using a Siemens 128-slice CT scanner with a standard protocol. Acquisition parameters included a tube voltage of 120 kV and a current of 100–200 mA; slice thickness of 1 mm; and interslice spacing of 1 mm. Bone algorithm reconstructions were generated with a lung window setting. Images were acquired at full inspiration, covering the entire thoracic area, with the patient in the supine position. Additional prone slices were obtained to rule out gravity-dependent changes. An experienced radiologist conducted both qualitative and quantitative analyses. Qualitative image assessment focused on identifying elementary lesions suggestive of ILD, including reticulations, fissural distortion, septal thickening, traction bronchiectasis, honeycombing, and ground-glass opacities. The chest HRCT pattern was categorized based on lesion type and distribution as either nonspecific interstitial pneumonia (NSIP) or usual interstitial pneumonia (UIP) (14, 15).

Quantitative analysis of ILD was performed using the Warrick semi-quantitative scoring system, which takes into account both the severity and the extent of interstitial involvement (16). A Warrick score of 0 corresponds to a normal appearance. Interstitial involvement is considered mild for scores below 8, moderate for scores between 8 and 15, and severe for scores above 15. We used a Warrick score threshold of 7 to define significant interstitial involvement. This threshold served as the dichotomous outcome measure for analyzing the diagnostic performance of LUS in detecting ILD (17).

### Statistical analysis

Data analysis was performed using the Statistical Package for the Social Sciences (IBM SPSS Statistics, version 25). Quantitative variables with a normal distribution were expressed as mean ± standard deviation, while non-parametric quantitative variables were presented as median and interquartile range. Qualitative variables were expressed as frequencies and relative percentages. Comparisons of means between groups were conducted using the student’s *t*-test for normally distributed variables. Non-parametric tests were applied for variables that did not follow a normal distribution. A *p*-value ≤ 0.05 was considered statistically significant. Spearman’s correlation coefficient (*r*) was used to assess the relationship between lung ultrasound data and continuous variables. Receiver Operating Characteristic (ROC) curve analysis was performed to evaluate the diagnostic performance of LUS in predicting interstitial involvement, defined dichotomously based on chest HRCT results. Sensitivity, specificity, positive predictive value (PPV), and negative predictive value (NPV) of LUS were calculated using an online calculator (18).

### Ethics approval statement

This study received a favorable opinion from the Local Committee for the Protection of Persons (CLPP) at Hôpital Militaire de Tunis (Meeting No. 68, Decision No. 32/2023/CLPP, dated June 19, 2023). The committee, coordinated by Prof. Haroun Ouertani (Head of Endocrinology-Nutrition Service), reviewed the protocol for the study titled “Early diagnosis of rheumatoid lung: contribution of thoracic ultrasound.”

### Consent procedure

Written informed consent was obtained from all participants prior to their inclusion in the study, in accordance with CLPP requirements and prevailing Tunisian ethical standards. Participants were informed of the study’s objectives, procedures, potential risks, and their rights, including the right to withdraw at any time without prejudice.

## Results

### Patients’ clinical characteristics

**Table 1:** Demographic, clinical, and treatment characteristics of RA patients evaluated for the role of LUS in diagnosing ILD in RA.

Parameters	Frequency (%)
Body mass index >= 25 Kg/m^2^	47 (64.4)
Active smoker	8 (11)
Positive rheumatoid factor (RF)	63 (86.5)
Positive anti-CCP antibodies	64 (87.7)
Positive antinuclear antibodies	22 (30.1)
DAS28-ESR >= 5.1	43 (58.9)
csDMARDs*	67 (91.8)
Anti-TNFα therapy**	16 (30)
Rituximab	11 (15.1)
Tocilizumab	3 (4.1)
Chronic cough	2 (2.7)
Dyspnea	12 (12.4)
Digital clubbing	1 (1.3)
Desaturation during the 6MWT	2 (2.7)
Restrictive ventilatory defect	7 (5.11)

*csDMARD: Methotrexate, Leflunomide, Sulfasalazine, Hydroxychloroquine; **Anti-TNFα agents, Infliximab: Etanercept, Certolizumab, Adalimumab.

BMI, body mass index; Kg, killogram; m, meter; LUS, lung ultrasound; RA, rheumatoid arthritis; 6MWT, 6 min walk test.

A total of 118 patients with RA presented to our rheumatology department for their routine follow-up of rheumatic disease. After screening for eligibility and exclusion criteria, 73 patients were included and completed the study, the study flowchart is presented in Figure 1.

**Figure 2:**
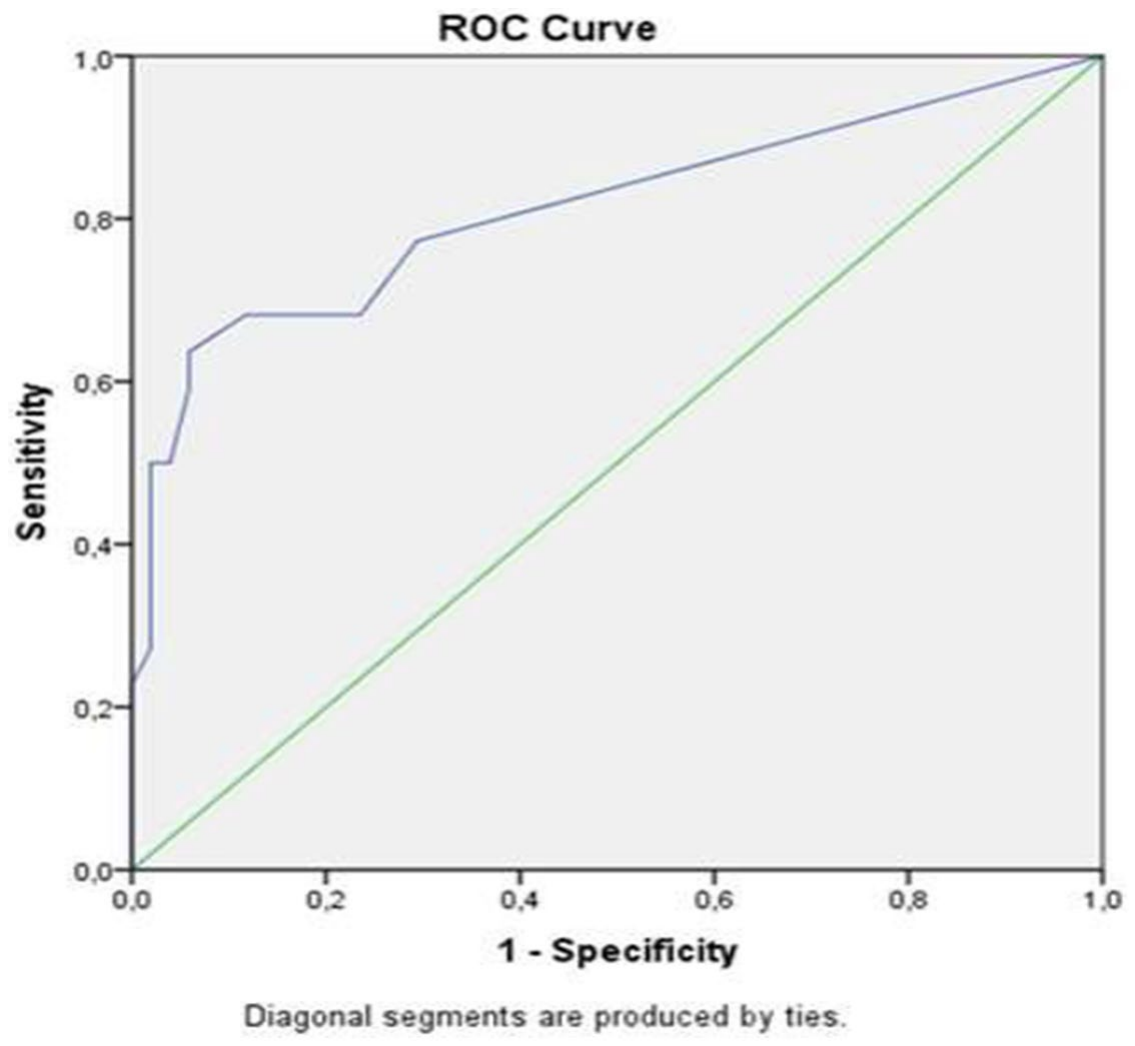
The study population flowchart.

Among the 73 patients, there were 18 men and 55 women. The mean age was 55 ± 12 years [range: 26–81]. The mean body mass index (BMI) was 26.51 ± 4.53 kg/m^2^. The average disease duration was 8.48 ± 7.96 years. The mean Disease Activity Score based on 28 joints using erythrocyte sedimentation rate (DAS28-ESR) was 3.47 ± 1.40. Inflammatory markers showed a median erythrocyte sedimentation rate (ESR) of 12 mm/h [interquartile range, IQR: 4.5–32] and a median C-reactive protein (CRP) level of 8 mg/L [IQR: 0–20.5]. Pulmonary function tests revealed a median forced expiratory volume in 1 s (FEV₁) of 102% predicted [IQR: 100–104], a median forced vital capacity (FVC) of 99% predicted [IQR: 90.5–101], and a median FEV₁/FVC ratio of 101% [IQR: 96–110]. Main demographic, clinical, and treatment characteristics of the enrolled patients are summarized in Table 1.

### Chest HRCT findings

Chest HRCT identified pulmonary emphysema in 9.6% of patients (*n* = 7) and ILD in 28.8% (*n* = 21). Among those with interstitial abnormalities, a typical nonspecific interstitial pneumonia (NSIP) pattern was observed in 9.6% of cases, and a cryptogenic organizing pneumonia (COP) pattern in 4.1% (*n* = 3). In 15.1% of patients, the distribution and nature of the elementary lesions did not correspond to any specific interstitial pattern. Ground-glass opacities were the most frequently observed elementary lesion, reported in 19.2% of patients (*n* = 14). Other radiological findings included traction bronchiectasis (15.1%), reticular opacities (15.1%), septal thickening (13.7%), pulmonary fibrosis (12.3%), honeycombing (9.6%), and fissural distortion (4.1%). The median Warrick score was 3 ± 7, ranging from 0 to 27. Significant interstitial involvement, defined as a Warrick score ≥ 8, was identified in 30.1% of patients (*n* = 22).

### LUS findings

Pleural irregularities were observed in 12.3%, while B-lines were present in 41.1% of patients. Notably, all patients with pleural irregularities had more than five B-lines. According to the semi-quantitative ultrasound score, interstitial involvement was detected in 21.9% of patients (*n* = 16), including mild involvement in 8.2% (*n* = 6), moderate in 11% (*n* = 8), and severe in 2.7% (*n* = 2).

### Correlation between LUS and chest HRCT findings

A statistically significant association was demonstrated between ILD patterns on chest HRCT and the following ultrasound abnormalities: B-lines, pleural irregularities, and a B-line count > 5 (Table 2).

**Table 2:** Association between LUS abnormalities and chest HRCT findings in RA patients.

LUS findings		Pleural Irregularity: No *n* (%)	Yes *n* (%)	B-lines: No *n* (%)	Yes *n* (%)	B-lines >5: No *n* (%)	Yes *n* (%)
HRCT findings							
NSIP pattern	No	61 (95.3)	**5 (55.6)** **	42 (97.7)	*24 (80)* *	56 (98.2)	**10 (62.5)** **
	Yes	3 (4.7)	**4 (44.4)** **	1 (2.3)	*6 (20)* *	1 (1.8)	**6 (37.5)** **
COP pattern	*No*	*55 (85.9)* *	*4 (44.4)* *	39 (90.7)	*20 (66.7)* *	*49 (86)* *	*10 (62.5)* *
	Yes	9 (14.1)	5 (55.6)	4 (9.3)	10 (33.3)	8 (14)	6 (37.5)
Ground-glass opacity	No	**57 (89.1)** **	**2 (22.2)** **	**41 (95.3)** **	**18 (60)** **	**53 (93)** **	**6 (37.5)** **
	Yes	7 (10.9)	**7 (77.8)** **	2 (4.7)	**12 (40)** **	4 (7)	**10 (62.5)** **
Honeycombing	No	**62 (96.9)** **	**4 (44.4)** **	**43 (100)** **	**23 (76.7)** **	**57 (100)** **	**9 (56.3)** **
	Yes	2 (3.1)	**5 (55.6)** **	0 (0)	**7 (23.3)** **	0 (0)	**7 (43.8)** **
Traction bronchiectasis	No	**60 (93.8)** **	**2 (22.2)** **	**42 (97.7)** **	**20 (66.7)** **	**55 (96.5)** **	**7 (43.8)** **
	Yes	4 (6.3)	**7 (77.8)** **	1 (2.3)	**10 (33.3)** **	2 (3.5)	**9 (56.3)** **
Septal thickening	No	**61 (95.3)** **	**2 (22.2)** **	**42 (97.7)** **	*21 (70)* *	**55 (96.5)** **	**8 (50)** **
	Yes	3 (4.7)	**7 (77.8)** **	1 (2.3)	*9 (30)* *	2 (3.5)	**8 (50)** **
Fissural distortion	*No*	*63 (98.4)* *	*7 (77.8)* *	43 (100)	*27 (90)* *	57 (100)	*13 (81.3)* *
	Yes	1 (1.6)	2 (22.2%)	0 (0%)	3 (10%)	0 (0%)	3 (18.8%)
Reticulations	No	**59 (92.2)** **	*3 (33.3%)* *	**41 (95.3%)** **	*21 (70%)* *	**54 (94.7%)** **	**8 (50%)** **
	Yes	5 (7.8)	*6 (66.7%)* *	2 (4.7%)	*9 (30%)* *	3 (5.3%)	**8 (50%)** **
Fibrosis	No	**62 (96.9)** **	**2 (22.2%)** **	**43 (100%)** **	**21 (70%)** **	**57 (100%)** **	**7 (43.8%)** **
	Yes	2 (3.1)	**7 (77.8)** **	0 (0%)	**9 (30%)** **	0 (0%)	**9 (56.3)** **

*p < 0.05; ** *p* < 0.001. Bold values indicate *p* < 0.001.

LUS, lung ultrasound; HRCT, high-resolution computed tomography; RA, rheumatoid arthritis; *n*, number; %, percentage; NSIP, non-specific interstitial pneumonia; COP, cryptogenic organizing pneumonia.

A moderate to strong positive correlation was observed between the total number of B-lines on LUS and the Warrick score (*r* = 0.685; *p* < 0.01).

### Diagnostic accuracy of LUS for detecting ILD in RA

To evaluate the diagnostic accuracy of LUS, the number of patients with and without ILD detected by LUS was compared to those with or without involvement on chest HRCT, considered as the reference standard (Table 3). The sensitivity of LUS was 59.1% (95% CI: 36.35–79.29), and the specificity was 94.12% (95% CI: 83.76–98.77). The positive predictive value (PPV) was 98.27% (95% CI: 94.74–99.45), while the negative predictive value (NPV) was 28.88% (95% CI: 19.65–40.26).

**Table 3:** Comparison of LUS and HRCT findings for the detection of ILD.

Present	HRCT: No ILD *n* (%)	HRCT: ILD *n* (%)	Total *n* (%)
No ILD on LUS	48 (94.1)	9 (40.9)	57 (78.1)
ILD on LUS	3 (5.9)	13 (59.1)	16 (21.9)
Total	51 (100)	22 (100)	73 (100)

ILD, interstitial lung disease; LUS, lung ultrasound.

The diagnostic performance of B-line count varies notably depending on the cut-off value used. A threshold of ≥1 B-line yields a high sensitivity of 77.3% but a moderate specificity of 70.6%. Increasing the cut-off to ≥5 B-lines significantly improves specificity to 94.1%, although sensitivity decreases to 63.6%. At a threshold of ≥7 B-lines, specificity further increases to 96.1%, with a corresponding drop in sensitivity to 50.0%. Finally, a cut-off of ≥19 B-lines provides maximal specificity (100%) but with very low sensitivity (22.7%). These findings suggest that an intermediate threshold, such as ≥5 B-lines, may offer a reasonable diagnostic balance, maintaining high specificity while preserving acceptable sensitivity.

ROC curve analysis demonstrated an area under the curve (AUC) of 0.813 (Figure 2), indicating good diagnostic performance of LUS in detecting ILD. This performance was statistically significant (*p* < 0.001), with a 95% confidence interval ranging from 0.690 to 0.936. Analysis of the ROC curve coordinates identified the optimal cutoff at 5 B-lines, yielding a sensitivity of 63.6% and a specificity of 94.1%. The highest sensitivity (77.3%) was observed at a threshold of ≥1 B-line, whereas the highest specificity (100%) was reached at a threshold of ≥19 B-lines.

## Discussion

The emergence of antifibrotic agents has improved the prognosis of RA-associated ILD when treated early (19). Thus the implementation of evidence-based, radiation-free, and accessible diagnostic tools to enable early diagnostic workup is justified. This study evaluated the diagnostic performance of LUS in RA-associated ILD. To achieve this goal, and in the absence of a consensus LUS protocol, we used the 12-zone protocol previously applied to monitor pulmonary changes in idiopathic pulmonary fibrosis. Our results, consistent with prior studies, demonstrated a strong correlation between semi-quantitative LUS score and chest HRCT score (12).

Our study demonstrated a statistically significant association between the presence of ILD on chest HRCT and the following LUS findings: pleural irregularities, B-lines, and a B-line count >5. A significant correlation was also observed between interstitial patterns on HRCT and these specific sonographic abnormalities. These findings are consistent with previously published data, particularly the results reported by Buda et al. (20). The analysis of correlations between interstitial abnormalities on HRCT and LUS findings in patients with RA revealed that reticulations on chest HRCT showed the strongest correlation with LUS abnormalities, particularly B-lines and irregular or thickened pleural lines. Honeycombing was moderately associated with several LUS features, especially confluent B-lines forming a “white lung” appearance and pleural irregularities. Similar findings were reported in the study by Sofíudóttir et al. (21). A moderate to strong positive correlation was also observed between the total number of B-lines on MUS and the Warrick score. This finding aligns with existing literature: studies conducted in RA patients have shown a statistically significant correlation between B-line counts and the Warrick score (22).

The study conducted by Cogliati et al. (23) also reported a positive correlation between the severity of ILD on HRCT and the number of B-lines observed on LUS (*p* < 0.05, *r* = 0.8).

Indeed, LUS does not provide a direct anatomical representation of the pulmonary parenchyma; rather, it detects ultrasound artifacts that reflect alterations limited to the peripheral subpleural space (24). It allows the visualization of ILD through the detection of B-lines vertical, hyperechoic comet-tail artifacts that originate from the pleural line and extend to the bottom of the screen without fading. These artifacts (25) reflect increased density of the pulmonary interstitium due to inflammatory, edematous, or fibrotic changes within the lung parenchyma (26). However, LUS does not allow differentiation between recent edematous changes and chronic fibrotic lesions (27), Moreover, LUS does not enable the identification of other pulmonary manifestations that may occur in RA, such as rheumatoid nodules (20).

In our study, LUS demonstrated a moderate sensitivity of 59.1% (95% CI: 36.35–79.29) for the detection of ILD. Comparable results were reported by Natalia Mena-Vázquez et al., who also found a moderate sensitivity of 62.2% in detecting early interstitial abnormalities. Their study, which included 71 RA patients divided into case and control groups, involved ultrasound examination of 72 intercostal spaces (25). This diagnostic performance of LUS could be influenced by the characteristics of the studied population, particularly a high BMI: 47% of patients included in our study were obese. Excess subcutaneous adipose tissue can indeed limit the penetration of ultrasound beams, thereby reducing image quality. Furthermore, the presence of pulmonary emphysema, identified in 9.6% of our patients (*n* = 7), may act as a confounding factor in the interpretation of ultrasound artifacts. In this context, alveolar destruction, altered pulmonary architecture, and decreased parenchymal density may impair the recognition of characteristic ultrasound signs of ILD (28).

The moderate sensitivity of LUS for detecting ILD does not allow exclusion of this condition in patients with a negative LUS. In such cases, it is advisable to look for other indicators suggestive of ILD that would justify performing a diagnostic chest HRCT scan, notably by calculating the risk score for ILD in asymptomatic patients (29), Look for respiratory symptoms, perform PFT, and request a chest X-ray.

Our study demonstrated a high specificity of 94.12% (95% CI: 83.76–98.77), supporting the reliability of LUS in confirming interstitial involvement. Similarly, Natalia Buda et al. reported a comparably high specificity of 91.3%, further reinforcing the consistency of LUS performance across different patient populations (30). The high specificity of LUS justifies further chest HRCT in ultrasound-positive patients to assess the extent and severity of ILD (31).

Interestingly, 5.9% of patients (*n* = 3) showed ILD on lung ultrasound without corresponding ILD on chest CT. Indeed, in autoimmune patients, sonographic changes in the latero-basal areas of the lung parenchyma and simultaneously at the pleura can occur even if chest HRCT appears normal (32).

We identified a cutoff of 5 B-lines as the best compromise between sensitivity and specificity. In the literature, a similar cutoff was reported in the study by Mena-Vázquez et al. (27), where 71 RA patients were recruited using an 8-zone LUS. In contrast, in the study by Di Carlo et al. (33). Seventy-one RA patients were recruited, and a 14-zone lung ultrasound protocol was used, with a B-line cutoff of 9. Indeed, the cutoff varies depending on the sites and the number of intercostal spaces examined.

Our study has several strengths to consider, we used a LUS protocol that is not time-consuming (12 intercostal zones), reliable, and reproducible. It ensures an equitable assessment across the different anterior, lateral, and posterior thoracic zones on both sides (12), these zones have been validated as predictive of changes related to ILD (8). It has been validated for the assessment of pulmonary changes in idiopathitic pulmonary fibrosis (12). Furthermore, this study validated LUS as a relevant diagnostic tool for detecting ILD in RA patients. It enables effective triage of patients for chest HRCT, reserving it for cases requiring detailed assessment of lesion severity and extent or to confirm a positive diagnosis.

The main limitations of this study are the high proportion of obese patients and the presence of pulmonary emphysema in some participants, which may impair ultrasound beam propagation and complicate the interpretation of sonographic artifacts, thus necessitating cautious analysis of the results. Additionally, the maximum interval of up to 300 days between lung ultrasound and HRCT in some cases could be a potential confounding factor; however, the median interval was 30 days, and patients with longer intervals showed stable respiratory symptoms, minimizing this risk. Finally, the sample size was limited by consecutive recruitment over a 6-month period, which may have affected the balance between patients with and without interstitial involvement but reflects the real clinical setting.

Based on these findings, we recommend integrating LUS into the diagnostic work-up of ILD during RA follow-up. Chest HRCT should complement LUS in the presence of sonographic abnormalities specifically pleural irregularities or >5 B-lines, to assess the extent and severity of ILD. Given the moderate sensitivity of LUS, particularly in obese patients or those with emphysema, a negative result does not rule out ILD; therefore, clinical and functional respiratory assessment, along with ILD risk score calculation, remains essential to guide the need for diagnostic HRCT. In addition, we advocate for broader training in LUS and the standardization of examination protocols. Future prospective multicenter studies are needed to validate the diagnostic performance of LUS in RA-ILD and to establish standardized scanning approaches, especially regarding the number of zones explored and the optimal B-line threshold. The development of composite LUS scoring systems incorporating B-line count, distribution, and pleural abnormalities may further enhance diagnostic precision and support risk stratification for fibrosing progression.

## Conclusion

Our study demonstrates that LUS findings of ILD in RA correlate well with chest HRCT data. ROC curve analysis revealed good diagnostic performance of LUS for ILD detection, characterized by moderate sensitivity but excellent specificity, allowing stratification of chest HRCT indications based on LUS results.

## Data Availability

The raw data supporting the conclusions of this article will be made available by the authors, without undue reservation.

## Glossary

STE: Speckle-tracking echocardiography
SpA: Spondyloarthritis
RA: Rheumatoid arthritis
ILD: Interstitial lung disease
PFT: Pulmonary function testing
HRCT: Chest high-resolution computed tomography
LUS: Lung ultrasound
6MWT: 6-min walk test
ROC: Receiver Operating Characteristic
BMI: Body mass index
DAS28-ESR: Disease Activity Score based on 28 joints using erythrocyte sedimentation rate
ESR: Erythrocyte sedimentation rate
CRP: C-reactive protein
FEV₁: Forced expiratory volume in one second
FVC: Forced vital capacity
NSIP: Nonspecific interstitial pneumonia
COP: Cryptogenic organizing pneumonia
PPV: Positive predictive value
NPV: Negative predictive value
AUC: Area under the curve
